# Initiation of opiate addiction in a Canadian prison: a case report

**DOI:** 10.1186/1477-7517-3-11

**Published:** 2006-03-16

**Authors:** Evan Wood, Ronald Lim, Thomas Kerr

**Affiliations:** 1 British Columbia Centre for Excellence in HIV/AIDS, St. Paul's Hospital, 608 – 1081 Burrard Street, Vancouver, BC V6Z 1Y6, Canada; 2 Department of Medicine, University of British Columbia, 950 West 10th Avenue, Vancouver, BC V5Z 4E3, Canada; 3 Addiction Centre, University of Calgary, 6th Floor, North Tower, 1403 – 29 Street NW, Calgary AB T2N 2T9, Canada

## Abstract

**Background:**

In North America, the harms of illicit drug use have been responded to primarily through law enforcement interventions. This strategy has resulted in record populations of addicted individuals being incarcerated in both Canada and the United States. The incarceration of non-violent drug offenders has become increasingly controversial as studies demonstrate the harms, including elevated HIV risk behavior, of incarcerating injection drug users. Other harms, such as the initiation of illicit drug use by prison inmates who previously did not use drugs, have been less commonly described.

**Case Presentation:**

We report on the case of an individual who initiated non-injection opiate use in a Canadian prison and developed an addiction to the drug. Upon release into the community, the individual continued using opiates and sought treatment at a clinic. The patient feared that he might initiate injection use of opiates if his cravings could not be controlled. The patient was placed on methadone maintenance therapy.

**Conclusion:**

While anecdotal reports indicate that initiation in prison of the use of addictive illicit substances is frequent, documentation through clinical experience is rare, and the public health implications of this behavior have not been given sufficient attention in the literature. Strategies of incarcerating non-violent drug offenders and attempting to keep illicit drugs out of prisons have not reduced the harms and costs of illicit drug use. Effective, practical alternatives are urgently needed; expanded community diversion programs for non-violent drug offenders deserve particular attention.

## Background

In North America, policy-makers have primarily responded to the public health emergency among injection drug users (IDU) by allocating resources to criminal justice interventions. The reliance on law enforcement has resulted in record incarceration rates in the United States and Canada. [1] The incarceration of illicit drug users also has major implications for public health because of the potential for infectious disease transmission in prison. [2-5] This may be of particular concern for human immunodeficiency virus (HIV) transmission, which has been previously documented among inmates in a Scottish prison [4,6] and suspected in several other settings as a result of syringe sharing between incarcerated injection drug users. [7-9]

The concentration of addicted individuals within prisons is believed to result in elevated HIV risk behavior because incarcerated IDU generally have high rates of HIV and HCV, and the interventions that are available in the community, such as well managed agonist detoxification and maintenance treatment and needle exchange, are often not available in prison. [10] In addition to syringe sharing, there are a number of other potential harms of mixing addicted populations with other inmates. Here, we report on a case of an opiate-addicted individual seeking methadone maintenance therapy (MMT) in the community soon after his release from a Canadian prison.

## Case presentation

A 20-year-old white male was seen as an outpatient at an addiction medicine clinic in Calgary, Canada. The male reported alcohol and cocaine use prior to being arrested, and the patient acknowledged that chaotic behavior related to the use of these substances had contributed to his crimes and eventual incarceration. While incarcerated, the individual reported initiating use of illicit (prescription) opiates, and his preferred route of administration was via nasal inhalation. Although the patient reported that his fellow inmates had frequently injected (he even reported assisting with injections), the patient denied injection use of illicit drugs in prison. The patient reported experiencing severe physical withdrawal symptoms during periods of non-opiate use in prison and soon became a regular user.

Upon release into the community, the patient reported that he had continued his xuse of diverted pharmaceutical opiates and that he had developed a pattern of use consistent with severe physical addiction. At the time of presentation at the clinic, the patient's urine tested positive for opiates. At that time, the patient reported that his unstable condition was such that he feared he might initiate intravenous opiate injection use if he did not do something to control his opiate cravings. The patient did not say why he had not injected opiates in prison but reported recognizing that injection opiate use would represent a further negative progression of his opiate addiction. After some discussion, the patient was started on methadone maintenance therapy, 10 mg per day, increasing 10 mg per day until desired therapeutic effect was reached.

## Conclusion

We have described a case of an individual initiating opiate addiction in a Canadian prison. With respect to this issue, it is important to stress that initiation of the use of addictive illicit substances while incarcerated has been previously reported. For instance, one study reported that 20% of IDU surveyed initiated injection drug use in prison. [11] However, while this is believed to occur frequently (from anecdotal reports), it is rarely documented through clinical experience. Accordingly, the public health and social implications of initiation of illicit drug addiction in prison have not received significant attention in the literature. This is of concern, since this issue has major implications for both prison and community health.

To date, the principal strategy to address initiation of illicit drug use in prison has been to redouble efforts to keep illicit drugs out of the prisons, though correction officials concede that this is almost impossible. However, over the last several decades, experience has proven that more pragmatic solutions are available and can be effective. [10,12] One pragmatic strategy has been the provision of methadone maintenance therapy within the prison system in an effort to reduce demand for illicit opiates. Unfortunately, however, this strategy is likely to be insufficient (e.g., for those who are primarily stimulants users – a growing population), and it is noteworthy that there was still illicit opiate use occurring in the prison where the patient described in this report initiated his opiate use, even though methadone was available there. While the expansion and evaluation of addiction treatment measures in prisons is an immediate priority, the burgeoning levels of incarceration of drug users and the rates of ongoing illicit drug use among incarcerated individuals indicate that additional strategies must be considered. [1-4,6-9] This implies that alternative programs to incarceration, such as the treatment of addiction in the community, may be more effective at reducing the health and social harms and economic costs of illicit drug use. [13,14] Expanded addiction services in prison should therefore be coupled with expansion and evaluation of community diversion programs for non-violent drug offenders.

In this brief report, we have described a case of an individual initiating opiate use and developing dependency to opiates within a Canadian prison. Unfortunately, this case is not unique, [11] and the concerns it raises are relevant to the many illicit drug users who do not use opiates in the community but risk initiating opiate use (including intravenous use) when incarcerated. And although the risks of HIV transmission in prison have been widely reported, the implications of initiation of illicit drug addiction in prison have not received adequate attention by public health and correctional policy-makers. Effective practical interventions are needed to address this concern, including expansion of strategies involving expanded community diversion programs for non-violent drug offenders. These deserve particular attention, given their potential to reduce HIV risk for current injectors who are incarcerated and to reduce the prevalence of initiation of illicit drug use (and, potentially, injecting) in the non-addicted inmate population.
